# The cerebrospinal fluid Neutrophil gelatinase‐associated lipocalin (NGAL) concentration in Alzheimer’s Disease (AD)

**DOI:** 10.1002/alz.088677

**Published:** 2025-01-09

**Authors:** Julia Doroszkiewicz, Agnieszka Kulczynska‐Przybik, Renata Borawska, Jan Mroczko, Agnieszka Slowik, Barbara Mroczko

**Affiliations:** ^1^ Department of Neurodegeneration Diagnostics, Medical University of Bialystok, Bialystok Poland; ^2^ Department of Neurology, Jagiellonian University, Krakow Poland; ^3^ Department of Biochemical Diagnostics, Medical University of Bialystok, Bialystok Poland

## Abstract

**Background:**

Alzheimer’s disease (AD), a prevalent neurological illness, is the most common form of dementia worldwide. Given that AD symptoms manifest gradually, it is imperative to discover new biomarkers that support early diagnosis of this diseases. Some research indicates that Neutrophil gelatinase‐associated lipocalin (NGAL) may influence a number of neurobiological processes, including inflammation occurring in the central nervous system. Moreover, this protein was discovered in brains of AD patients. Literature data suggests that NGAL could be a potential biomarker of severity of Alzheimer’s disease. Therefore, the purpose of our research was to assess the usefulness of the measuring of the concentration of soluble NGAL in cerebrospinal fluid in the diagnosis of AD patients as well as compare it with classical AD biomarkers.

**Method:**

The concentrations of NGAL were measured in cerebrospinal fluid (CSF) of 20 AD patients and 20 non‐demented controls using ELISA method. Classical biomarkers, such as Aβ‐42, Aβ‐42/Aβ‐40, tau and pTau181 were assessed by immunoenzyme assays.

**Result:**

NGAL concentrations were significantly higher in AD patients in comparison to non‐demented controls. Additionally, increased CSF levels of NGAL correlated positively with Tau and pTau181 proteins as well as negatively with Aβ‐42/Aβ‐40 ratio in the whole study group.

**Conclusion:**

Findings of our research suggest a potential role of NGAL in pathology of AD. However, follow‐up studies on larger study group are needed.

